# Perfluoroalkyl-Functionalized Hyperbranched Polyglycerol as Pore Forming Agents and Supramolecular Hosts in Polymer Microspheres

**DOI:** 10.3390/ijms160920183

**Published:** 2015-08-26

**Authors:** Olaf Wagner, Maximilian Zieringer, Wynter J. Duncanson, David A. Weitz, Rainer Haag

**Affiliations:** 1 Institute for Chemistry and Biochemistry, Freie Universität Berlin, Takustrasse 3, Berlin 14195, Germany; E-Mail: olaf.wagner@fu-berlin.de; 2 School of Engineering and Applied Sciences, Harvard University, 29 Oxford St., Cambridge, MA 02138, USA; E-Mails: mzieringer@hotmail.com (M.Z.); wynter.duncanson@nu.edu.kz (W.J.D.); weitz@seas.harvard.edu (D.A.W.); 3 Department of Physics, Harvard University, 29 Oxford St., Cambridge, MA 02138, USA

**Keywords:** polyglycerol, non-covalent, fluorous, supramolecular, microbubbles, microfluidics, porous, template, functional, host-guest system

## Abstract

Perfluoroalkyl-functionalized, hyperbranched polyglycerols that produce stable microbubbles are integrated into a microfluidic emulsion to create porous microspheres. In a previously-presented work a dendrimer with a perfluorinated shell was used. By replacing this dendrimer core with a hyperbranched core and evaluating different core sizes and degrees of fluorinated shell functionalization, we optimized the process to a more convenient synthesis and higher porosities. The new hyperbranched polyglycerol porogens produced more pores and can be used to prepare microspheres with porosity up to 12% (*v*/*v*). The presented preparation forms pores with a perfluoroalkyl-functionalized surface that enables the resulting microspheres to act as supramolecular host systems. The microspheres can incorporate gases into the pores and actives in the polymer matrix, while the perfluoroalkylated pore surface can be used to immobilize perfluoro-tagged molecules onto the pores by fluorous-fluorous interaction.

## 1. Introduction

Porous particles are versatile biomedical tools for the encapsulation, transport, and sustained release of drugs [1], gases [2], catalysts, enzymes [3], and cells [4]. In general, such particles are synthesized through emulsification of a matrix polymer, actives, and pore-forming agents, or porogens. Usually, pores are created in a subsequent step after particle formation. Various methods have been developed and combined to introduce porosity in microparticles, such as emulsion templating [5], freeze-drying [6], and particle leaching [7]. Unfortunately, employing homogenizers, glass membranes, ultrasound, or harsh leaching processes to create porous particles can lead to microspheres with broad morphological distributions, as well as low encapsulation efficiencies [8]. These limitations can be overcome by microfluidic emulsification techniques; they allow the controlled production of monodisperse microspheres with high encapsulation efficiency. Moreover, the removal of microsphere material to create pores can be avoided by using permanent template porogens that do not require leaching to produce pores [9,10,11].

Perfluorocarbons (PFCs) phase separate from water, as well as most organic solvents, due to their extremely low intermolecular attractive forces. This hydro- and lipophobicity leads to self-assembly in liquids and preferential organization at the liquid-gas interface of molecules with perfluorinated moieties [12]. Highly-fluorinated amphiphiles have been employed to stabilize micrometer-size bubbles for ultrasound imaging or oxygen delivery [13,14]. In aqueous media fluorinated amphiphiles self-organize by non-covalent fluorous-fluorous interaction. Moreover, these unique physicochemical properties can be utilized for the immobilization of perfluoro-tagged substrates on perfluorinated surfaces like microarrays [15], flourous silica gel [16], or dendrimers with perfluoroalkyl shells [17].

Previously, we have presented polyglycerol dendrimers (dPG) with perfluoroalkylated shells as a self-organizing, permanent template. It has been demonstrated that these dendrimers stabilize gas bubbles in the micrometer range in water and organic solvents [18]. Additionally, these stabilized gas bubbles not only serve as permanent geometric templates in the production of porous polymer microspheres but also confer additional functionality to the surface pores of the particles. We have demonstrated that the resultant surface pores can immobilize additional guest compounds through fluorous-fluorous interactions and thus enable the fabrication of microspheres, which can release multiple actives independently [18].

Typically, dendrimers are prepared through a multi-step synthesis, which involves several tedious purification steps. This synthetic pathway results in perfectly branched molecules (degree of branching 100%) with a spherical morphology [19]. Prominent alternatives to dendrimers are hyperbranched polymers which show a lower degree of branching and, therefore, less regular architectures [20]. These polymers, with their multitude of branches, are created in a one-pot synthesis without the necessity of multiple synthetic steps required for traditional dendrimer synthesis. Hyperbranched polymers are widely used in applications that do not depend on the monodisperse molecular weight and spherical morphology of dendrimers [21]. Hyperbranched polyglycerols (hPGs) are prepared through a ring-opening multi-branching polymerization (ROMBP) that yields randomly branched hPGs in a single-step synthesis and a degree of branching of 60% [22,23]. Whereas dPG can be functionalized via the hydroxyl groups on the dendrimer surface, hPG allows functionalization of the hydroxy groups on the surface as well as throughout the random polymer scaffold [24]. Therefore, hPG functionalized with perfluoroalkyl chains result in core-shell particles with a more random morphology than perfluoroalkylated dPG dendrimers. These perfluoroalkylated hPG amphiphiles (F-hPGs) with their higher structural flexibility in comparison to their dendrimer analogs could improve their functionality at the liquid-gas interphase. Thus, perfluoroalkylated hPG (F-hPG) could stabilize a higher amount of gas microbubbles in solution than a spherical perfluoroalkylatd PG dendrimer (F-dPG) and, therefore, be a more efficient pore forming template.

In this study we present perfluoroalkylated hPG as a self-organizing, permanent template for fabricating porous microspheres. Hyperbranched polyglycerols with perfluoroalkylated shells (F-hPG) form supramolecular complexes with perfluoro-tagged disperse red (F-DR) that are soluble in DCM. These complexes stabilize gas bubbles in the micrometer range and are used as templates in microfluidic fabrication of porous microspheres. The resulting pore surfaces are tested for immobilizing a perfluoro-tagged fluorescein isothiocyananate (F-FITC) as model active by fluorous-fluorous interaction to demonstrate the suitability of the microspheres as carrier for perfluoro-tagged compounds.

## 2. Results and Discussion

To study the relation between polymer size and pore formation, we synthesized two hyperbranched polyglycerols with molecular weights of 7.5 and 13 kDa, respectively. Consecutively, we attached perfluoroalkyl chains to the polymers to form hPG cores with perfluorinated shells. These core-shell structures were prepared through a two-step protocol [25] based on allylation of the hydroxy groups, followed by radical addition of perfluorinated thiols to the resulting allyl moieties, as shown in Figure S1. To determine the effect of the perfluoroalkyl chain density in the shell on the formation and stability of microbubbles, we synthesize fully as well as partially functionalized polymers. By ^1^H-NMR measurements we determined the degree of functionalization (DF) of 100% for both the 7.5 kDa polyglycerol (7.5 kDa_100_) and the 13 kDa polyglycerol (13 kDa_100_), respectively. In addition, we partially functionalized the smaller hPG to obtain a DF of 50% (7.5 kDa_50_).

**Figure 1:**
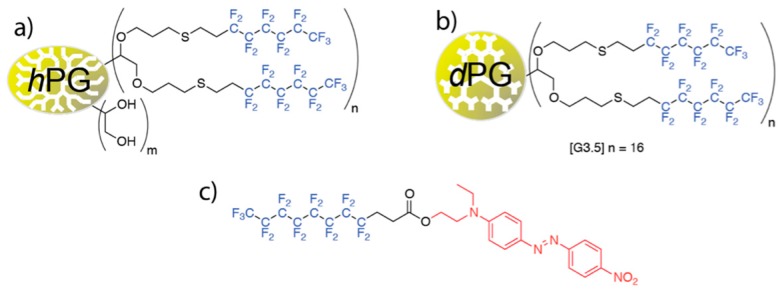
(**a**) Sketch of hyperbranched polyglycerol (hPG) with various degrees of perfluorinated alkyl functions (F-hPG); (**b**) Sketch of a perfectly branched perfluorinated PG dendrimer (F-dPG) where *n* = 4 for [G1.5], and *n* = 16 for [G3.5]; (**c**) Sketch of perfluoro-tagged Disperse Red 1 dye (F-DR).

Hyperbranched polyglycerols with perfluorinated shells (Figure 1) are not very soluble in organic solvents such as chloroform or dichloromethane [26]. Addition of a solubility mediating amphiphile, perfluoro-tagged disperse red (F-DR), to hPGs with perfluorinated shells leads to formation of supramolecular complexes which increases the solubility of these polymers in organic solvents. The increased solubility allowed the resulting supramolecular complexes to stabilize gas microbubbles in organic solvents [27].

We studied the ability of hPGs with perfluorinated shells to stabilize gas microbubbles in the presence of the matrix-forming polymer and evaluated their utility as a porogen for porous polymer microspheres. We generated these microbubbles by sonication of a solution that contained an equimolar ratio of hPG and perfluoro-tagged Disperse Red, Nile Red as contrast agent, and 5 wt % poly-lactic acid (PLA) as matrix-forming polymer in dichloromethane. As a comparison, we produced a sample with a [G3.5] PG dendrimer with perfluorinated shell, dPG_[G3.5]_, under the same experimental conditions. The following hPG subscript notation “*x* kDa *y*” describes the *M*_W_ of the hPG core (*x*) in kDa and the degree of shell fluorination (*y*) in percent of fluorinated OH groups.

**Figure 2:**
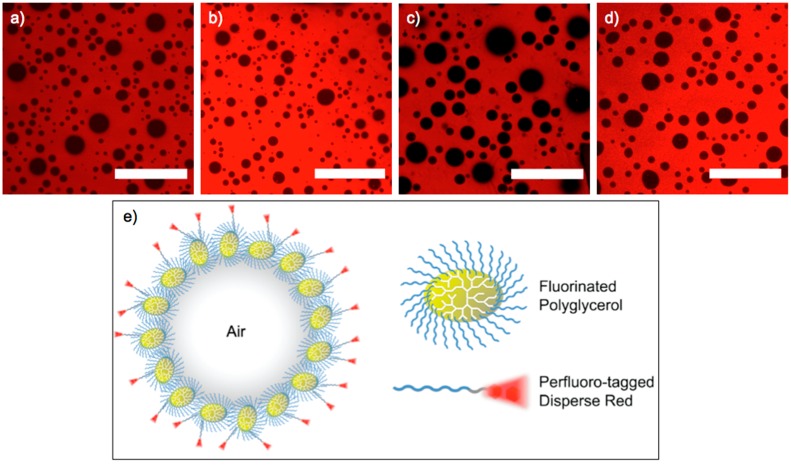
Fluorescence images of dichloromethane solutions containing Nile Red, PLA, and porogens: (**a**) hPG_7.5kDa100_; (**b**) hPG_13kDa100_; (**c**) dPG_[G3.5]_; and (**d**) hPG_7.5kDa50_. Scale bar: 50 µm. Microbubbles appear as black circles; and (**e**) Schematic of a stabilized microbubble formed through the self-assembly of F-hPG and F-DR.

The polyglycerols with perfluoroalkyl shell stabilized microbubbles with diameters in the range of 2.5 and 7 µm. We determined an average bubble diameter of 5.1 µm for hPG_7.5kDa100_, 4.1 µm for hPG_13kDa100_, and 7.0 µm for hPG_7.5kDa50_, respectively, as shown in Figure 2. For the dendrimer dPG_[G3.5]_, we observed microbubbles with an average diameter of 6.6 µm.

The amount of stabilized bubbles varied with the dendritic polymer employed. To qualitatively compare the amount of air stabilized by supramolecular complexes, we used confocal microscopy and viewed the top layer of a flat, predetermined volume of the sample solution. The volume fraction of non-fluorescent bubbles in the total volume was calculated. We determined a volume fraction of stabilized air of 31% for hPG_7.5kDa100_, 29% for hPG_7.5kDa50_, and 20% for hPG_13kDa100_, respectively. By contrast, the dendrimer dPG_[G3.5]_, stabilized a significantly smaller volume fraction of 16%, as summarized in Table 1.

**Table 1:** Overview of the different core type porogens and their air-in-solution values. All porogens were used at 1 mM concentration.

Name ^a^	Core Type	Bubble Number ^b^	Diameter (µm)	Air (%) ^c^
No porogen	None	5	0.9 ± 0.5	0.02 ± 0.01
[G3.5]	dPG	92	6.58 ± 2.49	15.6 ± 2.2
7.5kDa_100_	hPG	306	5.06 ± 3.24	30.8 ± 12.6
7.5kDa_50_	hPG	147	7.04 ± 2.84	28.6 ± 4.7
13kDa_100_	hPG	301	4.10 ± 2.34	19.9 ± 6.5

^a^ [G3.5] is a generation 3.5 PG dendrimer core with a perfluorinated shell. *x*kDa*_y_* describes hyperbranched PGs with a *M*_W_ of *x* kDa and *y* as the degree of shell fluorination in percent (OH groups fluorinated with C_f6_-chains); ^b^ Average number of bubbles counted in the screening area (20,000 µm^2^); and ^c^ Air-to-solvent ratio on screening area.

**Figure 3:**
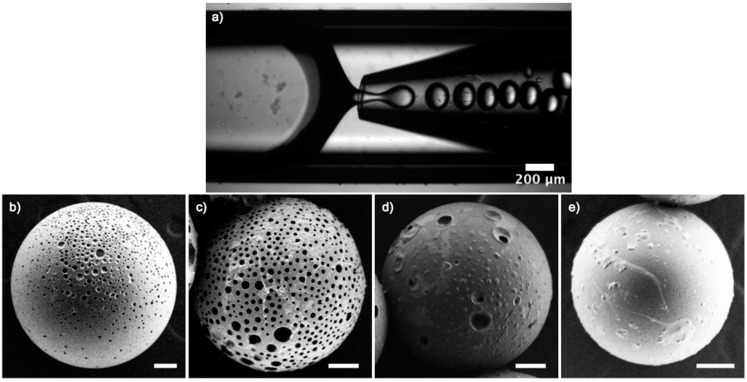
(**a**) Optical microscopy image of microfluidic droplet fabrication with (**b**–**e**). Scanning electron images of microspheres fabricated with porogens: (**b**) hPG_7.5kDa100_; (**c**) hPG_13kDa100_; (**d**) dPG_[G3.5]_; and (**e**) hPG_7.5kDa50_. Scale bar: 10 µm.

We fabricated polymer microspheres containing stabilized microbubbles in a microfluidic glass-capillary device to investigate the relation of the amount of stabilized air in solution to subsequent microsphere porosity. We used a device with flow-focusing geometry in which a round, tapered capillary was coaxially aligned within a square capillary. The dispersed phase, stabilized bubbles, Nile Red, and PLA in dichloromethane were injected into the device from one end of the square capillary while the continuous phase was injected from the opposite side (Figure S11) Monodisperse drops formed at the orifice of the round tapered capillary, as shown in Figure 3a. We produced drops containing either hPG_7.5kDa100_, hPG_13kDa100_, hPG_7.5kDa50_, or dPG_[G3.5]_ as the dendritic component of the supramolecular complexes. Subsequent solvent evaporation yielded monodisperse, porous PLA microspheres (Figure S12). We determined their porosity by confocal microscopy; we imaged the cross sections of the spheres to calculate the pore volume fraction (Supplementary information Section 5, Figure S13).

Our microfluidic strategy yielded microspheres with a pore volume of 12% for hPG_7.5kDa100_, 11% for hPG_13kDa100_, and 6% for dPG_[G3.5]_, respectively. By contrast, the microspheres produced with the partially functionalized hPG_7.5kDa50_ displayed almost no pores in the confocal measurements. We determined that only 0.5% of the total microsphere volume was occupied by pores. Only supramolecular complexes based on the fully functionalized dendritic polymers, hPG_7.5kDa100_, hPG_13kDa100_, and dPG_[G3.5]_, reliably served as a pore forming agent to produce porous PLA microspheres as shown in the SEM images in Figure 3.

This result indicates that a shell functionalization with perfluorinated chains of only 50% is not sufficient to maintain the bubble template structures. We assume that a degree of functionalization of only 50% was not sufficient to favor phase separation from the matrix forming PLA.

Both of the fully-functionalized, randomly-branched hPGs stabilize a significantly larger volume fraction of air bubbles and create microspheres with higher porosities than the perfectly branched dendrimer dPG_[G3.5],_ as summarized in Table 2.

**Table 2:** Overview of the porogens seen in Table 1 and their air-in-solution values compared to the resulting microsphere porosity.

Name ^a^	PG-Core Type	Air in Solution (%) ^b^	Air in Microsphere (%) ^c^
No porogen	None	0.02 ± 0.01	0.38 ± 0.21
[G3.5]	dPG	15.6 ± 2.2	5.72 ± 2.12
7.5kDa_100_	hPG	30.8 ± 12.6	12.10 ± 3.24
7.5kDa_50_	hPG	28.6 ± 4.7	0.44 ± 0.28
13kDa_100_	hPG	19.9 ± 6.5	11.03 ± 3.45

^a^ [G3.5] is a generation 3.5 PG dendrimer core with perfluorinated shell. *x*-kDa*_y_* describes hyperbranched PGs with a *M*_W_ of *x* kDa and *y* as the degree of shell fluorination in percent (OH groups fluorinated with C_f6_-chains); ^b^ Air-to-solvent ratio on screening area; and ^c^ Air-to-polymer matrix ratio in porous microsphere cross sections.

The less regular architecture of hyperbranched polyglycerols, due to their lower degree of branching compared to dendrimers, results in less spherical structures [20]. We attribute the higher ability to stabilize gas bubbles to the lower structural rigidity of hyperbranched polymers. We assume that the structural flexibility of these polymers may allow for an energetically favored orientation at the gas-solvent interphase; this may result in a higher amount of perfluoroalkyl chains oriented towards the gas phase.

**Figure 4:**
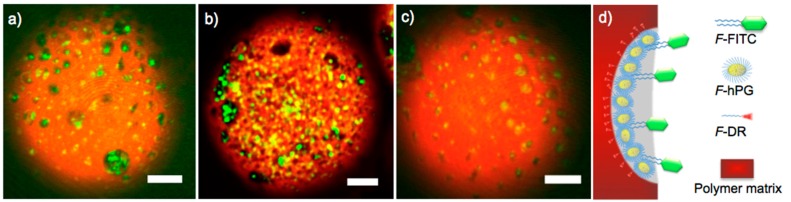
Fluorescence overlay images of bi-fluoro-tagged fluorescein (F-FITC) immobilized on microsphere pore surfaces created by porogens: (**a**) hPG_7.5kDa100_; (**b**) hPG_13kDa100_; (**c**) dPG_[G3.5]_. Scale bar: 10 µm; (**d**) schematic illustration of the immobilization of perfluoro-tagged FITC (F-FITC) by fluorous-fluorous interaction.

To evaluate the ability of hPG-based porogens to serve as binding site for perfluoro-tagged guest molecules, we attached bi-perfluoro-tagged fluorescein isothiocyanate (F-FITC) to the surface pores. The microspheres prepared with the porogens hPG_7.5kDa100_, hPG_13kDa100_, and dPG_[G3.5]_, respectively, were incubated with F-FITC in perfluorohexane and consecutively washed to remove any unbound F-FITC. We imaged each of the loaded red microspheres and found that the pore surfaces were coated by the green F-FITC, as shown in the red and green overlay images in Figure 4.

## 3. Experimental Section

### 3.1. Reagents and Materials

Commercially-available chemicals were purchased from reliable sources and used as delivered. Poly(dl-lactic acid) (PLA, *M*_W_ = 15,000 g·mol^−1^, Polysciences, Inc., Warrington, PA, USA) was used as the matrix-forming polymer for microspheres. Poly(vinyl alcohol) (PVA, *M*_W_ = 13,000–23,000 g·mol^−1^, 98% hydrolyzed, Aldrich, St. Louis, MO, USA) was used as the surfactant (5% *w*/*v*) for the outer aqueous phase. Nile Red was used as a hydrophobic dye for the inner oil phase. Dichloromethane (DCM, 99.8%, Mallinckrodt, Paris, KY, USA) served as an organic solvent for PLA and Nile Red. The square microcapillaries were purchased from Atlantic International Technologies (AIT, Rockaway, NJ, USA). The round glass microcapillaries were purchased from World Precision Instruments, Inc. (Sarasota, FL, USA) and tapered using a micropipette puller from Shutter Instruments Co. (Novato, CA, USA).

All aqueous solutions were filtered with Acrodisc 32 mm syringe filters and a 5 µm Supor membrane before use.

### 3.2. Syntheses of Perfluoroalkyl-Functionalized Compounds

DPG polyglycerol [G3] dendrimer and perfluoroalkyl functionalized dendrimer [G3.5] were prepared as described previously [18].

#### 3.2.1. Synthesis of Hyperbranched Polyglycerols (hPGs)

HPG cores of 7.5kDa_100_ and 13kDa_100_, respectively, were prepared through a one-step ring-opening anionic polymerization with trimethylolpropane (TMP) as initiator [22,23]. The monomer to initiator ratio for these hPGs was adjusted to yield an overall targeted molecular weight of *M*_n_ ≈ 7500 g·mol^−1^ and *M*_n_ ≈ 13,000 g·mol^−1^ with a PDI < 2.

#### 3.2.2. Synthesis of Perfluoroalkyl Functionalized Hyperbranched Polyglycerols (F-hPGs)

Allyl chloride, 5 equivalents (eq.) was added to a solution of hPG, tetrabutylammonium bromide (20 mol %) as a phase-transfer catalyst, and NaOH (5 eq.) in deionized water. The reaction mixture turned opaque and was stirred for 24 h at 50 °C. After extraction with toluene, the organic phase was separated, washed with brine, dried over MgSO_4_, filtered, and concentrated under vacuum. Further purification was achieved by dialysis in MeOH (2 times for 24 h with 2 kDa MWCO) to yield the desired product as a pale yellow oil (70% yield) (NMR spectrum in Figure S2). To receive 50% allylated hPGs as educts for 7.5 kDa_50_ 1.5 eq. of allyl chloride and NaOH were used. For long-term storage, it was necessary to keep the allyl ether product under an inert atmosphere at −20 °C. ^1^H-NMR (400 MHz, CDCl_3_, δ): 5.82–5.96 (m, 1H, CH), 5.26 (d, *J* = 18 Hz, 2H, CH_2_), 5.08–5.19 (m, 1H, CH_2_), 4.14 (d, *J* = 4 Hz, 1H, CH_2_), 3.99 (d, *J* = 4 Hz, 1H, CH_2_), 3.35–3.75 (m, 5H, CHO, CH_2_O) ppm. ^13^C-NMR (400 MHz, CDCl_3_, δ): 134.5 (allyl-CH), 117.6 (allyl-CH_2_), 65.0–81.5 (PG backbone) ppm.

A mixture of polyallyl polyglycerol and 3,3,4,4,5,5,6,6,7,7,8,8,8-tridecauorooctane-1-thiol (5 eq. to allyl groups) was stirred under reduced pressure (3 mbar) and repeatedly purged with argon to remove oxygen from the solution. After heating to 70 °C, azobisisobutyronitrile (AIBN) (0.5 eq.) was added under an argon atmosphere, and the reaction mixture was stirred for 2 h. After further addition of the same amount of AIBN, the mixture was stirred for another 20 h at 70 °C. The solvent was evaporated to yield a yellow product. Further purification was achieved by dialysis in trifluorotoluene (2 times for 24 h with 2 kDa MWCO) to yield the products as pale-yellow oil (98% yield) (NMR spectrum in Figures S3–S5). ^1^H-NMR (400 MHz, CDCl_3_, δ): 3.35–3.75 (m, 7H, CHO, CH_2_O), 2.62–2.78 (m, 4H, SCH_2_), 2.30–2.45 (m, 2H, CH_2_), 1.82–1.92 (m, 2H, CH_2_) ppm. ^13^C-NMR (400 MHz, CDCl_3_, δ): 109.0–120.0 (CF_2_), 64.8–81.0 (PG backbone), 32.6 (CH_2_CF_2_), 29.4 (SCH_2_), 22.9 (CH_2_) ppm. ^19^F-NMR (400 MHz, CDCl_3_ C_6_F_6_, δ): −81.3 (t, *J* = 10 Hz, CF_3_), −114.1 (CF_2_), −114.8 (CF_2_CF_3_), −122.3 (CF_2_), −123.3 (CF_2_), −123.9 (CF_2_), −126.7 (CF_2_) ppm.

#### 3.2.3. Synthesis of Perfluoro-Tagged Disperse Red 1 (F-DR)

A mixture of Disperse Red 1 and heptadecafluoroundecanoic acid (1 eq.) was dissolved in DCM. ECDI (2 eq.) and DMAP (1.2 eq.) were added to the solution and stirred for 72 h at RT. After the mixture was repeatedly washed with pure water, the solvent was removed under vacuum to yield a dark red residue. For further purification the raw product was washed with DMF and yielded the product as a red solid (41% yield) (spectra in Figures S6–S10). ^1^H-NMR (400 MHz, CDCl_3_, δ): 6.80 (d, *J* = 9.2 Hz , 8H, Ar-H) 4.35 (t, *J* = 6.0 Hz, 2H, CH_2_), 3.70 (t, *J* = 6.0 Hz, 2H, CH_2_), 3.52 (q, *J* = 7.2 Hz, NCH_2_, 2H,) 2.60–2.63 (m, 1H, CH_2_CH_2_CF_2_), 2.37–2.51 (m, 1H, CH_2_CH_2_CF_2_), 1.26 (t, *J* = 7.2 Hz, 3H, CH_3_) ppm.^13^C-NMR (400 MHz, CDCl_3_, δ): 171.2 (CO), 156.8 (CN), 151.3, 147.6, 144.0, 126.4 (CH), 124.8, 122.8, 111.6, 62.1 (NCH_2_CH_2_), 48.8 (NCH_2_CH_2_), 45.8 (NCH_2_CH_3_), 26.6 (COCH_2_CH_2_), 25.5 (COCH_2_CH_2_), 12.4 (CH_3_) ppm.^19^F-NMR (400 MHz, CDCl_3_, C_6_F_6_, δ): −80.6 (t, *J* = 10.4 Hz, CF_3_), −114.6 (CF_2_), 122.6 (CF_2_), −123.3 (CF_2_), −126.0 (CF_2_) ppm. MS (ESI-TOF): *m*/*z* = 789.14 [M + H]^+^.

### 3.3. Microsphere Fabrication

The microfluidic devices that were employed for the microsphere fabrication consisted of a glass slide, Teflon tubing (Scientific Commodities, Lake Havasu City, AZ, USA), a square glass capillary, two round tapered glass capillaries, and syringe needle tips. The device fabrication is further described Section 3 of the supplementary information (SI) and shown in Figure S11. The outer aqueous phase and inner oil phase were injected at independently adjustable flow rates using syringe pumps. An aqueous 5 wt % polyvinyl alcohol (PVA) solution was used as the outer aqueous phase and a 5 wt % polylactic acid (PLA) solution in DCM with Nile Red (0.1–2 mM) was used as the inner oil phase. The drops were collected in an aqueous 2 wt % PVA solution. Dichloromethane (DCM) was removed under reduced pressure and the dried spheres were washed with deionized water to remove residual PVA.

### 3.4. Microbubble and Microsphere Characterization

The amount of stabilized bubbles, as well as the resulting porosity and morphology of the microspheres, was characterized by optical and confocal microscopy and scanning electron microscopy (SEM). Optical and confocal microscopy images were obtained with a Leica TCS SP5 confocal microscope (Buffalo Grove, IL, USA). A sample chamber consisting of a microscope slide with an epoxy adhesive bonded plastic ring cut from a 0.5 mL Eppendorf tube was prepared, filled with the sonicated mixture of the porogen, PLA, DCM, and sealed with Parafilm^®^ to prevent solvent evaporation.

Samples of dried spheres were loaded on a double-sided carbon tape on an aluminum stud for SEM images and acquired with FESEM Supra55VP (Buffalo Grove, IL, USA)or an Ultra scanning electron microscope using secondary electron detection at 1 kV.

The average diameter and variation coefficient of the microbubbles was calculated from confocal microscopy images. For a matching refractional index the dried PLA microspheres were imaged in a formamide solution. The microscope focus was set to inside the microsphere in the *z*-direction to acquire cross section images. The cross sections showed a bright red area due to the Nile Red inside the PLA matrix and pores as black spots. To estimate the average porosity of the microspheres, multiple cross section images were viewed and calculated for their air (black) to PLA (red) ratio by ImageJ (National Institutes of Health, Bethesda, MD, USA). The average porosity was determined by calculating the porosity ratio of 20 cross sections of multiple microspheres randomly selected in each sample. The calculated porosities were compared to SEM imaged of the same batch with microspheres cut in half (Figure S12) and showed comparable porosities.

## 4. Conclusions

We synthesized various hyperbranched polyglycerols with perfluorinated shells that stabilize air bubbles in organic solvents upon formation of supramolecular complexes. Moreover, our polymers are excellent pore-forming agents that allow for the immobilization of functional compounds on the resulting pore surface.

Depending on the core and the degree of functionalization in the shells, a 1 mM solution of our polymers in dichloromethane contained up to 30% *v*/*v* of stabilized gas bubbles. This amount of stabilized gas represents a two-fold increase over the amount stabilized by polyglycerol dendrimers with perfluorinated shells. We attribute this result to the lower degree of branching of hyperbranched polymers and, therefore, to the higher structural flexibility of our core-shell compounds in comparison to perfectly branched dendrimers.

We used microfluidics to produce monodisperse porous PLA microspheres (Figure S13). By a comparison of the volume fraction of stabilized bubbles in solution to the resulting total pore volume of the spheres, we evaluated the ability of the hyperbranched core-shell structures to serve as permanent porogen. Both fully-functionalized polymers yielded pores upon solidification of the spheres with 11% volume fraction. Although the dendrimer control stabilizes a similar amount of gas in solution as the larger hPG, it yielded however only half the pore volume fraction in the solidified spheres. Furthermore, our results demonstrate that a high density of perfluorinated chains at the periphery of the polymers is crucial for yielding porous microspheres; the partially functionalized hPG does not yield pores upon solidification of the matrix polymer although it stabilizes a higher amount of gas in solution than the fully functionalized hPG.

The capability of hPG with perfluorinated shells to yield microspheres with an increased porosity, their facilitated synthesis, and their ability to immobilize perfluoro-tagged guest molecules should increase the value of dendritic polymers as permanent geometric template. In general, the aforementioned properties should make hyperbranched core-shell particles valuable for applications that require an independent controlled release of two actives. Our strategy to attach functional moieties to a bulk-synthesized core allows for future up scaling and implementation in high-throughput fabrication of functional porous microspheres.

## Supplementary Materials

Supplementary materials can be found at http://www.mdpi.com/1422-0067/16/09/20183/s1.
