# Supplementary material for: Perfluoroalkyl-Functionalized Hyperbranched Polyglycerol as Pore Forming Agents and Supramolecular Hosts in Polymer Microspheres
Source: Int J Mol Sci. 2015 Aug 26;16(9):20183–94. doi: 10.3390/ijms160920183 (PMC4613196; doi:10.3390/ijms160920183)
Supplement: Supplementary file 1 [file ijms-16-20183-s001.pdf]

Supplementary Information

1. Syntheses

1.1. Synthesis of Perfluoroalkyl Functionalized Hyperbranched Polyglycerol

Allyl chloride (15.3 g, 200 mmol) was added to a solution of polyglycerol (2.96 g, 40 mmol OH) tetrabutylammonium bromide (8 mmol, 20 mol %) as a phase transfer catalyst and NaOH (8 g, 200 mmol) in deionized water (8 mL). The reaction mixture changed color to a cloudy white and was stirred further for 24 h at 50 °C. After the addition of toluene, the organic phase was separated, washed with brine solution, dried over $MgSO_4$, filtered, and concentrated under vacuum. Further purification was achieved by dialysis in toluene to give the product as pale yellow oil (4.22 g, 69%). For long-term storage, it was necessary to keep the allyl ether product under an inert atmosphere at −20 °C.

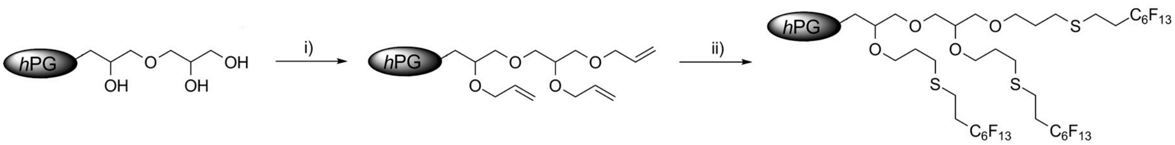

Figure S1. Perfluoroalkylation of hyperbranched polyglycerol hPG cores via allyl functionality and radical thiol addition: (i) Bu_4NBr, NaOH, allyl chloride, water, 24 h, 50 °C; (ii) $HSCH_2CH_2(CF_2)_5CF_3$, AIBN, 22 h, 70 °C.

^{1}H-NMR (400 MHz, $CDCl_3$, δ): 5.82–5.96 (m, 1H, CH), 5.26 (d, J = 18 Hz, 1H, CH_2), 5.08–5.19 (m, 1H, CH_2), 4.14 (d, J = 4 Hz, 1H, CH_2), 3.99 (d, J = 4 Hz, 1H, CH_2), 3.35–3.75 (m, 5H, CHO, CH_2O) ppm.

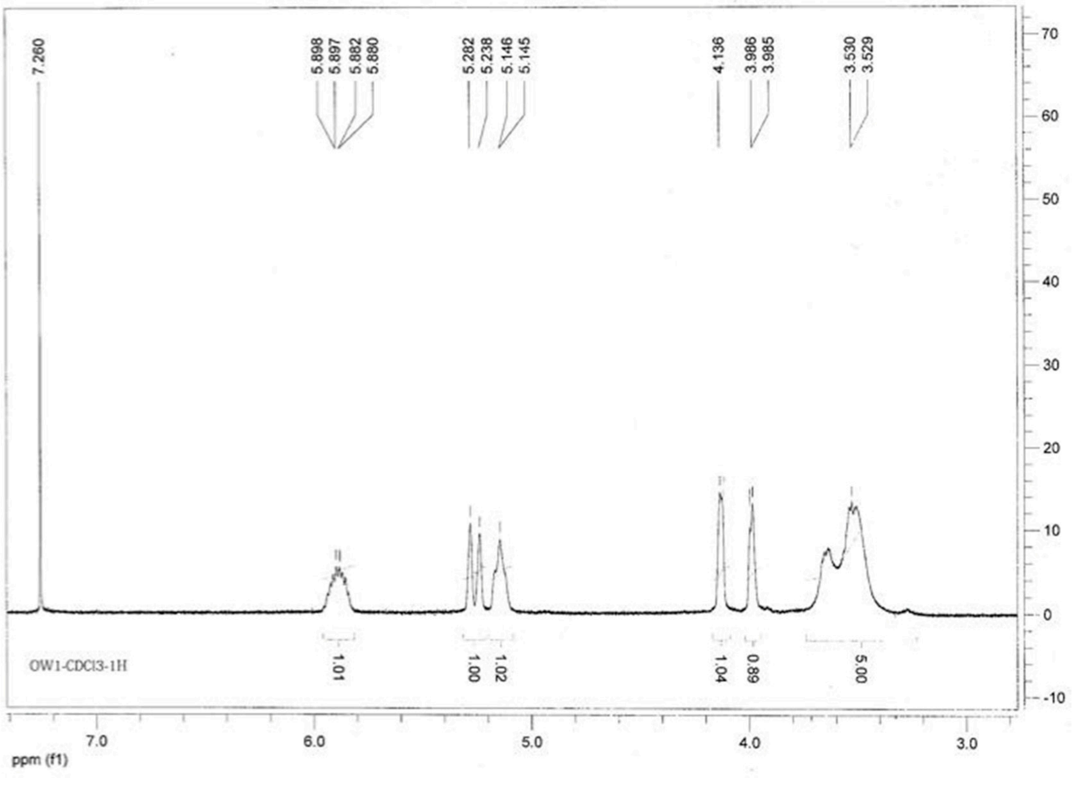

Figure S2. ^{1}H-NMR hPG-allyl.

A mixture of polyallyl polyglycerol (0.4 g, 3.5 mmol allyl groups) and 3,3,4,4,5,5,6,6,7,7,8,8,8-tridecauorooctane-1-thiol (6.66 g, 17.5 mmol) was stirred under reduced pressure (3 mbar) and purged with Argon repeatedly to remove oxygen from the solution. After heating to 70 °C, Azobisisobutyronitrile (AIBN) (11.5 mg, 0.07 mmol) was added under an atmosphere of Argon, and the reaction mixture was stirred for 2 h. After further addition of the same amount of AIBN, the mixture was stirred for another 20 h at 70 °C. The solvent was evaporated to yield a yellow product. Further purification was achieved by dialysis in trifluorotoluene (2 times for 24 h) to yield the product as pale-yellow Oil (1.71 g, 99%).

^{1}H-NMR (400 MHz, $CDCl_3$, δ): 3.35–3.75 (m, 7H, CHO, CH_2O), 2.62–2.78 (m, 4H, SCH_2), 2.30–2.45 (m, 2H, CH_2), 1.82–1.92 (m, 2H, CH_2) ppm.

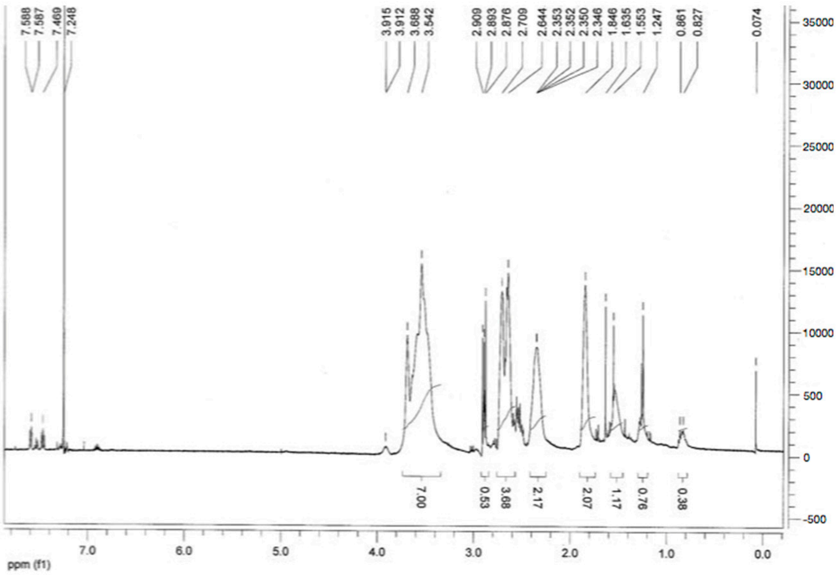

Figure S3. ^{1}H-NMR F-hPG.

^{13}C-NMR (400 MHz, $CDCl_3$, δ): 139.5–140.2 (CH_2O), 136.8–137.8 (CH_2O), 109.0–120.0 (CF_2), 32.6 (CH_2CF_2), 29.4 (SCH_2), 22.9 (CH_2) ppm.

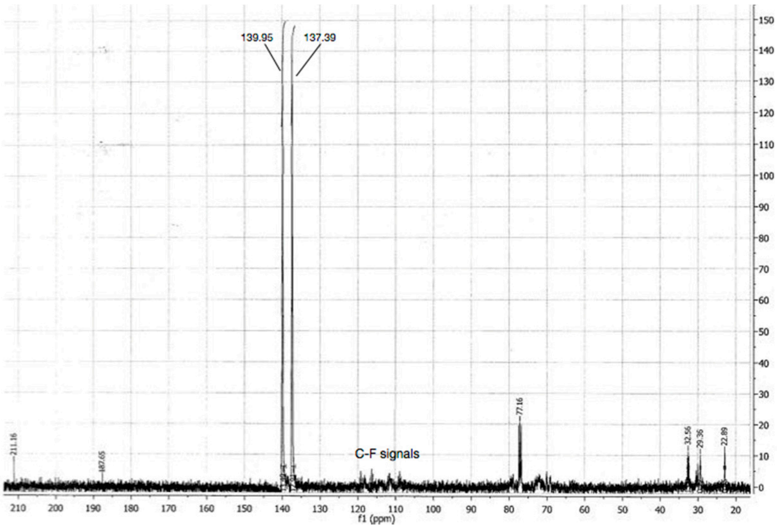

Figure S4. ^{13}C-NMR F-hPG.

^{19}F-NMR (400 MHz, $CDCl_3$ C_6F_6, δ): −81.3 (t, *J* = 10 Hz, CF_3), −114.1 (CF_2), −114.8 (CF_2CF_3), −122.3 (CF_2), −123.3 (CF_2), −123.9 (CF_2), −126.7 (CF_2) ppm.

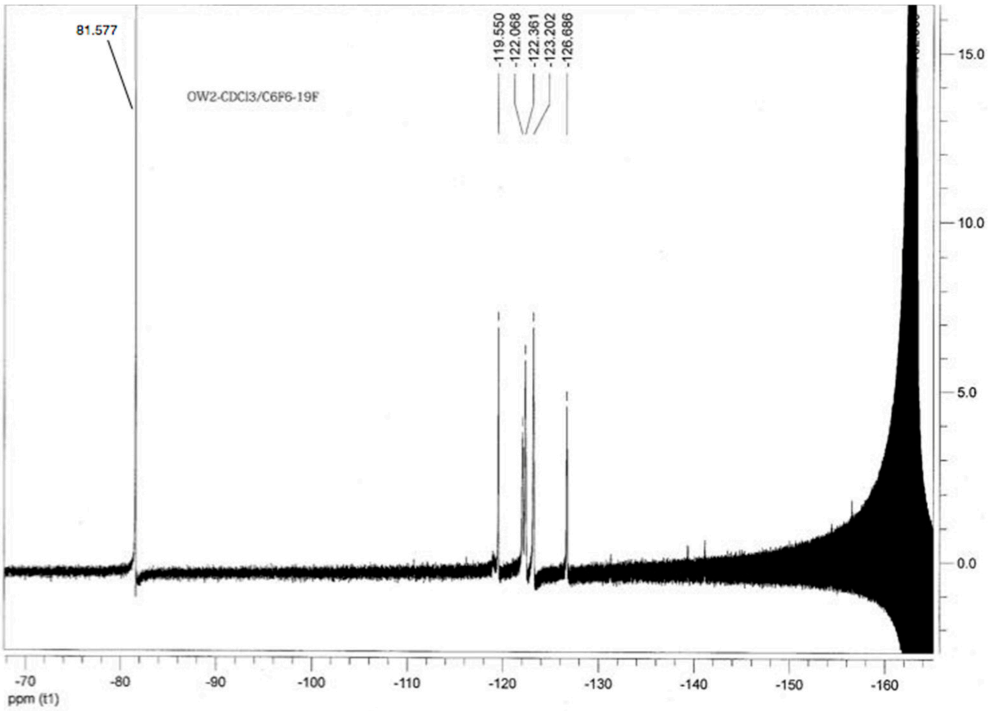

Figure S5. ^{19}F-NMR F-hPG.

IR: *ν* = 2920.0 (CH_2), 2867.9 (CH_2), 1824.8, 1362.5, 1326.0, 1232.6, 1186.4, 1142.1, 1119.4, 1069.6, 953.0, 845.5, 808.9, 772.0, 707.1 cm^{-1}.

1.2. Synthesis of Perfluoro-Tagged Disperse Red 1 (F-DR)

A mixture of Disperse Red 1 (426 mg, 1.36 mmol) and heptadecafluoroundecanoic acid (669 mg, 1.359 mmol) was dissolved in DCM (50 mL). ECDI (519 mg, 2.72 mmol) and DMAP (208 mg, 1.70 mmol) were added to the solution and stirred for 72 h at rt. After the mixture was repeatedly washed with pure water the solvent was removed under vacuum to yield a dark red residue. For further purification the raw product was washed with DMF and yielded the product as a red solid (540 mg, 50%).

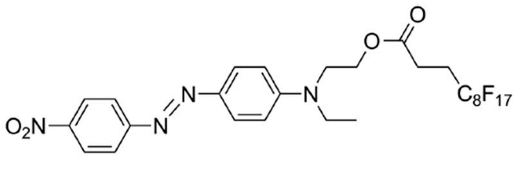

Figure S6. Chemical structure of perfluoro-tagged Disperse Red 1 (F-DR).

^{1}H-NMR (400 MHz, $CDCl_3$, δ): 6.80 (d, *J* = 9.2 Hz , 8H, Ar-H) 4.35 (t, *J* = 6.0 Hz, 2H, CH_2), 3.70 (t, *J* = 6.0 Hz, 2H, CH_2), 3.52 (q, *J* = 7.2 Hz, NCH_2, 2H,) 2.60–2.63 (m, 1H, $CH_2CH_2CF_2$), 2.37–2.51 (m, 1H, $CH_2CH_2CF_2$), 1.26 (t, *J* = 7.2 Hz, 3H, CH_3) ppm.

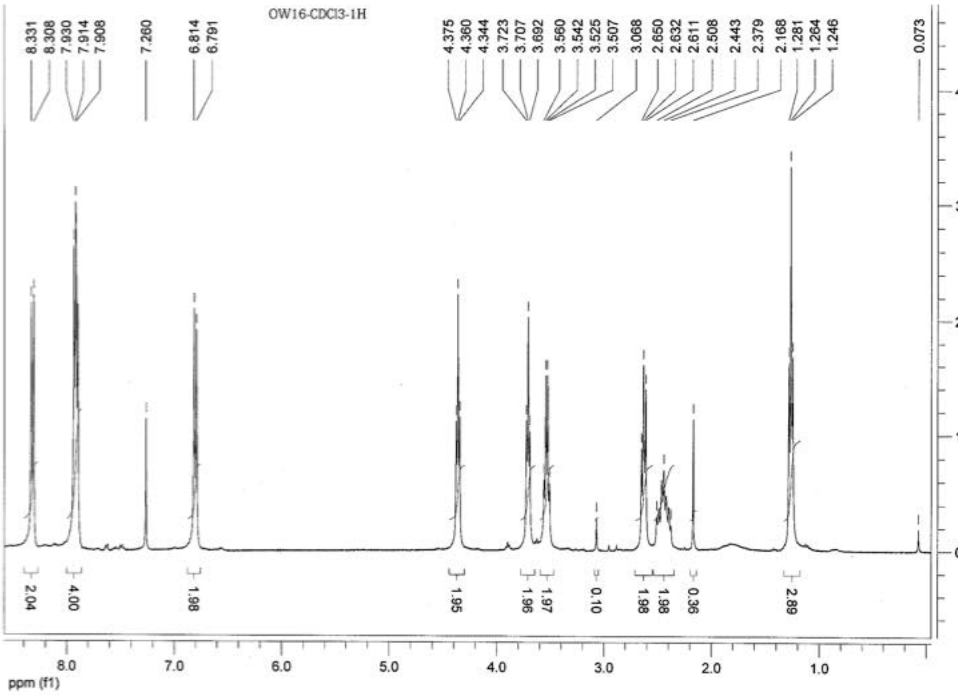

Figure S7. ^{1}H-NMR F-DR.

^{13}C-NMR (400 MHz, $CDCl_3$, δ): 171.2 (CO), 156.8 (CN), 151.3, 147.6, 144.0, 126.4 (CH), 124.8, 122.8, 111.6, 62.1 (NCH_2CH_2), 48.8 (NCH_2CH_2), 45.8 (NCH_2CH_3), 26.6 ($COCH_2CH_2$), 25.5 ($COCH_2CH_2$), 12.4 (CH_3) ppm.

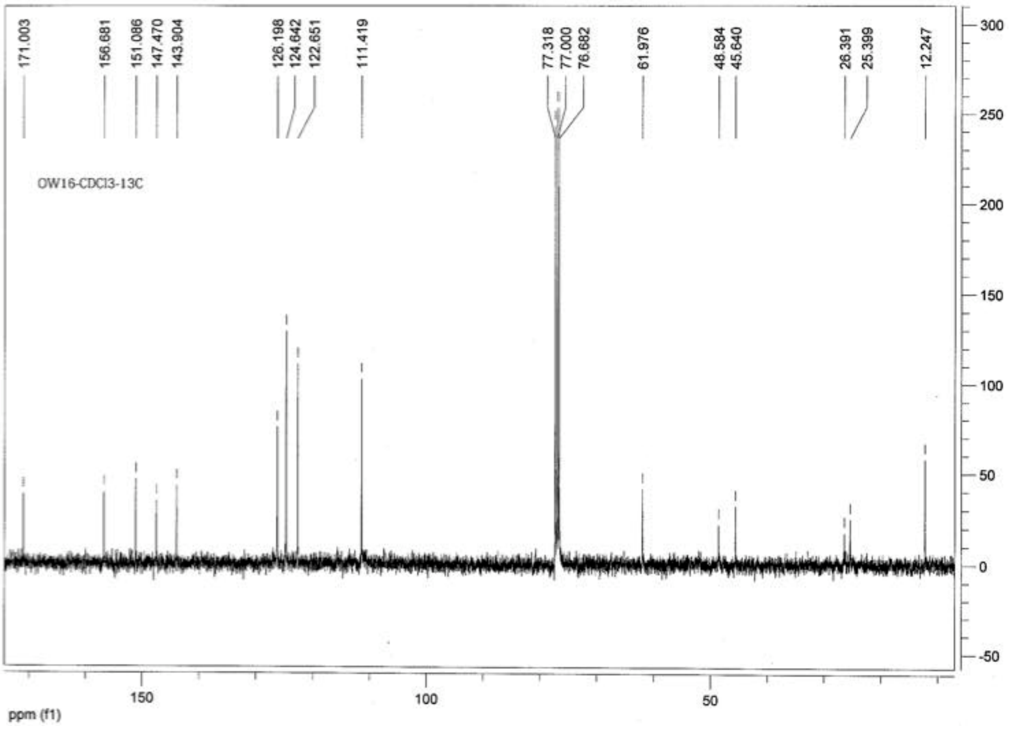

Figure S8. ^{13}C-NMR F-DR.

^{19}F-NMR (400 MHz, $CDCl_3$, C_6F_6, δ): −80.6 (t, J = 10.4 Hz, CF_3), −114.6 (CF_2), 122.6 (CF_2), −123.3 (CF_2), −126.0 (CF_2) ppm.

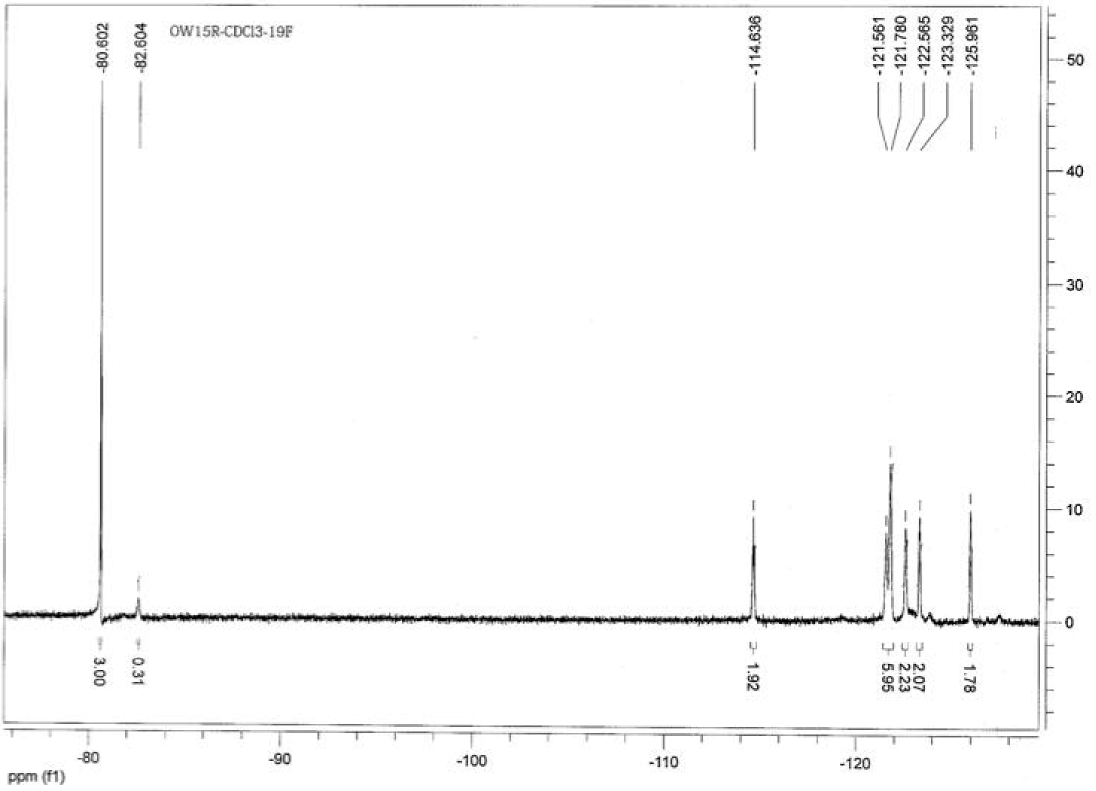

Figure S9. ^{19}F-NMR F-DR.

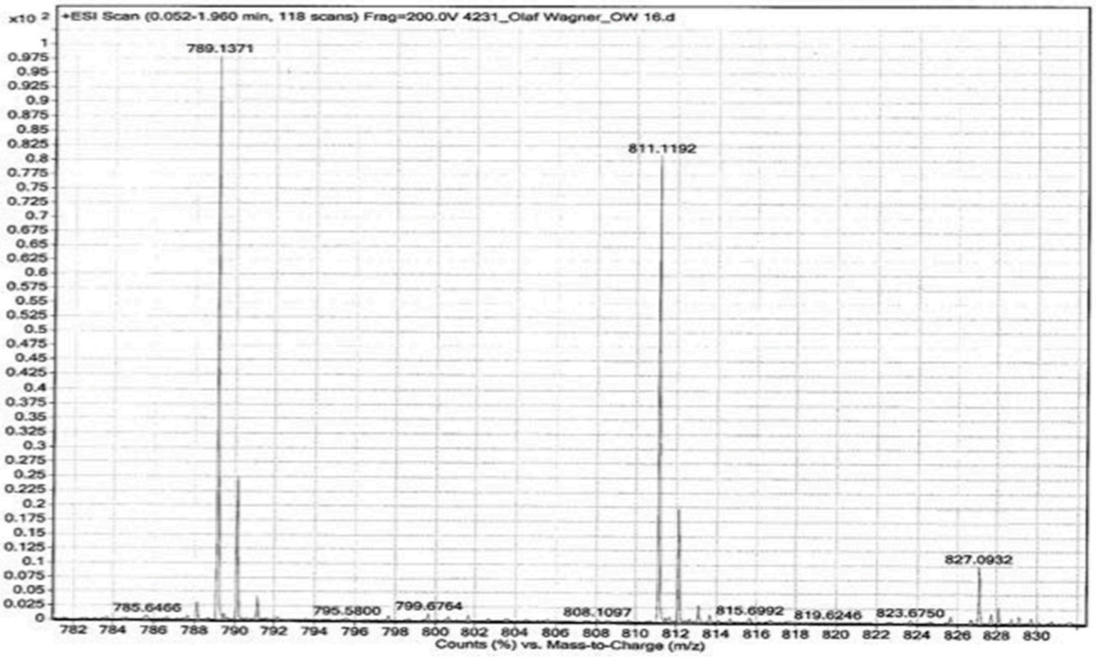

Figure S10. ESI-TOF F-DR.

MS (ESI-TOF): *m*/*z* = 789.14 $[M + H]^+$, 811.12 $[M + Na]^+$, 827.09 $[M + K]^+$. IR: v = 2979.1 (Ar-H), 1745.2 (C=O), 1599.3, 1513.5, 1388.4, 1338.2, 1196.7, 1134.0, 1010.7, 983.3, 858.0 and 825.3 cm^{-1}.

2. Materials

Commercially available chemicals were purchased from reliable sources and used as delivered. Poly(dl-lactic acid) (PLA, M_W = 15,000 g·mol^{-1}, Polysciences, Inc., Warrington, PA, USA) was used as matrix-forming polymer for microspheres. Poly(vinyl alcohol) (PVA, M_W = 13,000–23,000 g·mol^{-1}, 98% hydrolyzed, Aldrich) was used as surfactant (5% *w*/*v*) for the outer aqueous phase. Nile Red was used as a hydrophobic dye for the inner oil phase. Dichloromethane (DCM, 99.8%, Mallinckrodt) served as an organic solvent for PLA and Nile Red. The square microcapillaries were purchased from Atlantic International Technologies (AIT, Rockaway, NJ, USA). The round glass microcapillaries were purchased from World Precision Instruments, Inc. (Sarasota, FL, USA) and tapered using a micropipette puller from Shutter Instruments Co. (Novato, CA, USA). All aqueous solutions were filtered by Acrodisc 32 mm syringe filters with 5 µm Supor membrane before use.

3. Device Fabrication

The microfluidic devices that were used for the microsphere fabrication consisted of a glass slide, PE or Teflon tubes, two glass capillaries and a syringe tip. The device was fabricated by inserting the round capillary into the square capillary and mixing both with epoxy adhesive to the glass slide. Less than a centimeter of tube for the inner oil phase was inserted into the square capillary and sealed with epoxy glue. The direct tubing of the oil phase inlet without syringe tip avoids the formation of unwanted air reservoirs that cause pulsing and breaks of the continuous oil phase stream. To avoid wetting on glass surfaces by DCM, the capillaries were coated with the hydrophilic compound 2-[methoxy(polyethyleneoxy)-propyl]trimethoxysilane. The syringe tip was adjusting to the interconnection of the round capillary and the square capillary, followed by sealing with epoxy adhesive.

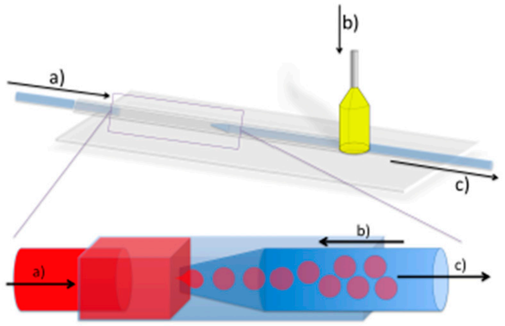

Figure S11. Schematic illustration of microfluidic glass capillary device. (**a**) inner oil phase: PLA solution (5 wt %) containing Nile red as fluorescent dye in DCM; (**b**) outer aqueous phase: PVA solution (10 wt %); and (**c**) resulting monodisperse droplets collected in aqueous 2 wt % PVA solution.

4. Microsphere Fabrication

The two phases—outer aqueous phase and inner oil phase—were infused at independently adjustable flow rates by syringe pumps connected to the device by tubing. An aqueous 10 wt % PVA solution was used as the outer aqueous phase and a 5 wt % PLA solution in DCM with Nile Red (0.1–2 mM) was

used as the inner oil phase. For the collection phase, an aqueous 2 wt % PVA solution was used. The collected DCM droplets in PVA solution were dried in at 700 mbar vacuum for 48 h to evaporate the DCM and to obtain solid microspheres. The spheres were washed with purified water to remove residual PVA.

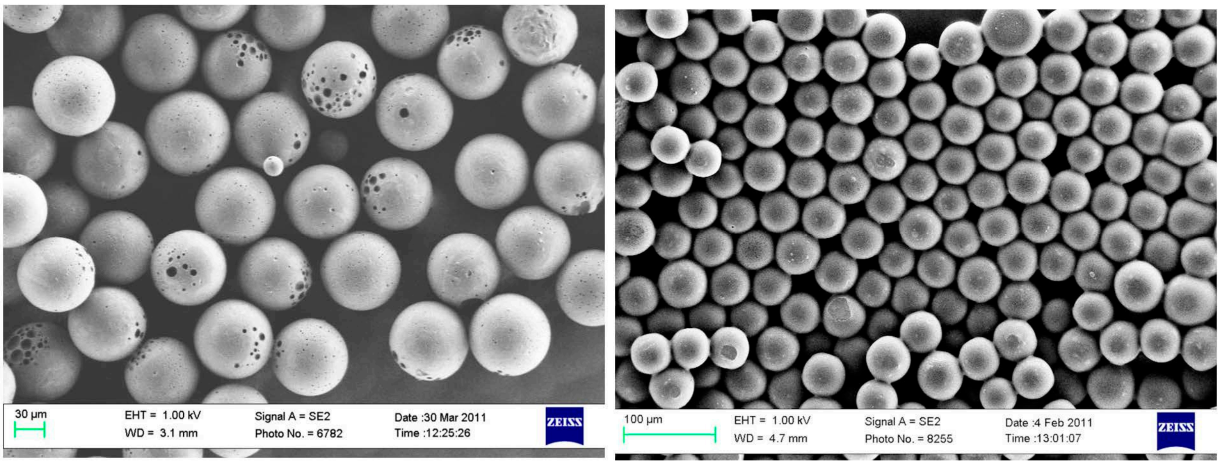

Figure S12. SEM pictures of multiple porous microspheres displaying their monodispersity.

5. Statistical Calculation of Diameters and Porosity

Number per area (20,000 µm^2), average diameter and coefficient of variation (CV) of the micron-sized bubbles were calculated from the confocal microscopy images. The value of CV is defined by CV = δ/[M] × 100, where δ is the standard deviation and [M] is the average diameter. The necessary contrast was created by using Nile Red in solution. Black air bubbles on red background were analyzed via picture threshold setting and particle measurement using ImageJ 1.0. Measurement values (sensitivity and range) were set to: circularity = 0.8–1, range = 0.5–5000 µm^2. The porosity of fabricated spheres was determined by cross section confocal images. The dried PLA particles were recorded in formamide for a matching refractional index and imaged in various depths within one sphere. The average porosity was determined by calculating the air to total volume ratio of 20 cross sections of multiple spheres randomly selected of each sample. Figure 4 shows an example of a cross section after area calculation. All measurements were done in triplicate.

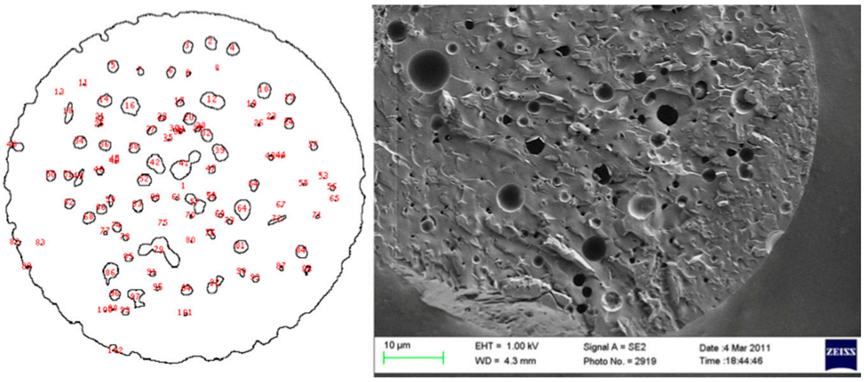

Figure S13. Example of a pore outlines image of PLA cross sections for porosity calculation (**left**), SEM picture of cross section of a porous sphere as comparison (**right**).
